# Evaluating strategies to recruit health researchers to participate in online survey research

**DOI:** 10.1186/s12874-024-02275-6

**Published:** 2024-07-18

**Authors:** Elizabeth R. Stevens, Charles M. Cleland, Amelia Shunk, Omar El Shahawy

**Affiliations:** 1 Department of Population Health, NYU Grossman School of Medicine, New York, NY USA; 2 School of Global Public Health, New York University, New York, NY USA

**Keywords:** Meta-research, Multiphase optimization strategy, Survey, Recruitment, Motivation, Health researchers

## Abstract

**Background:**

Engaging researchers as research subjects is key to informing the development of effective and relevant research practices. It is important to understand how best to engage researchers as research subjects.

**Methods:**

A 2^4^ factorial experiment, as part of a Multiphase Optimization Strategy, was performed to evaluate effects of four recruitment strategy components on participant opening of an emailed survey link and survey completion. Participants were members of three US-based national health research consortia. A stratified simple random sample was used to assign potential survey participants to one of 16 recruitment scenarios. Recruitment strategy components were intended to address both intrinsic and extrinsic sources of motivation, including: $50 gift, $1,000 raffle, altruistic messaging, and egoistic messaging. Multivariable generalized linear regression analyses adjusting for consortium estimated component effects on outcomes. Potential interactions among components were tested. Results are reported as adjusted odds ratios (aOR) with 95% confidence intervals (95% CI).

**Results:**

Surveys were collected from June to December 2023. A total of 418 participants were included from the consortia, with final analytical sample of 400 eligible participants. Out of the final sample, 82% (341) opened the survey link and 35% (147) completed the survey. Altruistic messaging increased the odds of opening the survey (aOR 2.02, 95% CI: 1.35–2.69, *p* = 0.033), while egoistic messaging significantly reduced the odds of opening the survey (aOR 0.56, 95%CI 0.38–0.75, *p* = 0.08). The receipt of egoistic messaging increased the odds of completing the survey once opened (aOR 1.81, 95%CI: 1.39–2.23, *p* < 0.05). There was a significant negative interaction effect between the altruistic appeal and egoistic messaging strategies for survey completion outcome. Monetary incentives did not a have a significant impact on survey completion.

**Conclusion:**

Intrinsic motivation is likely to be a greater driver of health researcher participation in survey research than extrinsic motivation. Altruistic and egoistic messaging may differentially impact initial interest and survey completion and when combined may lead to improved rates of recruitment, but not survey completion. Further research is needed to determine how to best optimize message content and whether the effects observed are modified by survey burden.

**Supplementary Information:**

The online version contains supplementary material available at 10.1186/s12874-024-02275-6.

## Background

Engaging researchers as research subjects is key to advancing the development of effective and relevant research practices. To promote robust science, meta-research (“research-on-research”) uses an interdisciplinary approach to examine research practices with the same scientific rigor given to other areas of scientific inquiry [1]. Within many meta-research themes, data on—and the perspectives of—researchers themselves have an important role to play for both identifying potential areas for improvement, as well as developing solutions to the problems identified [2]. Online survey research is a popular and valuable means of collecting research data [3]. However, successful recruitment of researchers to be participants in research can be a challenge, with participation rates often lower than among the general population [4–19]. 

Researchers often must balance many competing priorities with an overarching goal of research impact, [20] which can produce significant mental workload and contribute to burnout [21–28]. These activities may include teaching, funding acquisition, administrative duties, scholarly activities, and—for scholar clinicians—patient care [21–28]. Thus, the high workload and a pro-social (i.e. an intent to benefit others) motivation to ‘make a difference’ may indicate that researchers have differing incentives for, and barriers to, participating in research than the average population. Although survey recruitment has been the focus of much investigation in general, [29] and among health care providers, [30–36] there has been limited research performed on how best to recruit researchers—and health researchers more specifically—to engage in research as participants themselves. To increase representation and continue to advance meta-research, it is important to understand how best to engage researchers as research subjects.

To address this gap, the objective of this study was to identify the recruitment strategy components that impact health researcher participation rates in online survey research. In general populations, financial incentives have been found to enhance recruitment beyond that of altruistic incentives, [37, 38] however educated professionals have been shown to be less influenced by small monetary incentives [39] and more pro-social groups are potentially more incentivized by altruistic appeals than the general population [39, 40]. Due to their high workload, pro-social nature, and anecdotal reports that researchers will often leave even high-value monetary rewards ‘on the table,’ we hypothesized that appealing to a researcher’s altruism to help a fellow researcher and their personal interest in the survey topic would have a greater impact on survey participation than monetary incentives.

To test this hypothesis, we performed a randomized factorial experiment as part of the recruitment for the first wave of a longitudinal survey study of health researchers exploring collaborative research behaviors. The goal of using this factorial study approach was to delineate the impact of different engagement strategies on survey response to inform best messaging practice in subsequent follow-up surveys.

## Methods

We used a 2^4^ factorial experiment as part of a Multiphase Optimization Strategy (MOST) [41] to determine the recruitment strategy components most effective for engaging health researchers in an online survey among members of three research consortia. The use of a factorial experiment as part of the MOST framework allows investigators to assess the effect of each component of a program simultaneously with a much smaller sample size than would occur in randomized controlled trials for each strategy [41]. The experiment tested the effect of four distinct recruitment strategy components, as well as their interactions, on the opening and completion of the survey within the study population of health researchers. All study procedures were approved by the NYU Langone Health institutional review board (Study# 22-01099) who provided a waiver of informed consent for this study.

### Study setting and participants

Participants were members from three national health research consortia based in the United States. Research consortia are structured networks that bring together researchers from multiple institutions to participate in cooperative research efforts on particular focus areas. Health researchers were broadly defined as anyone who designated themselves as a researcher, and were not limited by academic degree (e.g. PhD, MD, etc.) or whether or not they were practicing clinicians. Participating consortia were selected based on convenience and consortium topical focus. Two consortia were based within the Veterans Health Administration, one focused on telehealth expansion (Consortium A) and the other focused on telehealth for cancer (Consortium B). The third one (Consortium C) was funded by the National Institutes of Health and focused on older adults with multiple chronic conditions. To improve survey relevance on the participant level, the Consortium C scholars’ program members were selected as a subset of the total Consortium C membership for participation. Potential survey participants were identified from consortium members on the consortium email distribution lists provided by the leadership of each consortium. To be included in the study an individual needed to: (1) have a valid email address on a consortium distribution list, (2) be a current consortium member, and (3) perform research activities. Those who perform only administrative functions (*n* = 18) within the consortium (e.g. administrative assistant on the mailing list to manage a calendar) were excluded from analyses rendering an analytical sample of *n* = 400. Excepting consortium affiliation, no demographic information was available regarding consortium participants prior to survey. The online survey was performed using Qualtrics’ XM platform [42] and took approximately 20 min on average to complete.

### Study design

In our 2^4^ factorial experiment, a stratified simple random sample was used to assign potential survey participants, based on the email distribution lists, to one of 16 recruitment scenarios that included a combination of standard messaging (as required by the institutional review board) and the four recruitment strategy components. Randomization was stratified by consortium; the randomization strategy was designed to ensure an equal number of participants assigned to each recruitment scenario within each participating consortium.

Potential participants were sent a survey recruitment email followed by follow-up reminder emails three times spaced approximately two weeks apart, or until the survey was completed, for a maximum of four email communications. All recruitment emails included components required by the ethics approval board including a study description, key participant information (e.g. voluntary nature of study), and a personalized survey link, as well as appropriate text for each recruitment scenario. The initial email and follow-up email text was identical outside of an introductory sentence included to indicate that the email was a follow-up from a previous email. It was not possible to determine which email wave led to clicking on the survey link.

### Recruitment strategy components

Potential survey participants received a randomly selected recruitment scenario that included a combination of standard messaging and up to four recruitment strategy components addressing factors associated with messaging content and incentives. Each recruitment strategy component had two levels, with a component only present or absent, producing a total of 16 combinations of recruitment components, including one scenario of standard messaging alone. Recruitment strategy components were intended to address both intrinsic and extrinsic sources of motivation, including: (1) standard messaging, (2) monetary gift ($50) incentive (extrinsic motivation), (3) monetary raffle ($1,000) incentive (extrinsic motivation), (4) personal appeal messaging (altruistic messaging), and (5) potential gain messaging (egoistic messaging). All potential participants received the standard messaging as part of the recruitment email. One-sixteenth (*N* ~ 26) of potential participants were randomly assigned to each of the 16 possible messaging combinations, including one scenario with the standard messaging alone (i.e., core component sent to all participants) and 15 recruitment strategy scenarios with a combination of at least one, and as many as four, recruitment components per scenario. Each recruitment component was present in eight of the 16 recruitment scenarios. Details of the component combinations included in the 16 recruitment strategy scenarios can be found in Supplement Table S1.

Standard messaging included an email with basic required study information including the study purpose and ethics disclosures, as well as one-time consortium leadership promotional messaging (i.e. an official notice from consortium leadership via separate email or newsletter that the survey will be occurring). The stated study purpose was for the survey more generally, and did not disclose the factorial study recruitment randomization. With the purpose of eliciting extrinsic motivation, two recruitment components provided monetary incentive for survey participation. The monetary gift recruitment component included the promise of a $50 gift certificate upon survey completion. The monetary raffle recruitment component included the opportunity to be entered in a drawing for a $1,000 check upon survey completion. The altruistic appeal messaging was designed to highlight the personal importance of survey participation to the survey investigator. The egoistic messaging was designed to highlight the potential (non-monetary) benefits to the participant for completing the survey. The recruitment email wording was held constant throughout each follow-up email, excluding the greeting, which was varied to inform potential participants of the follow-up nature of the email. See Supplement Table S2 for recruitment email wording associated with each recruitment strategy component.

### Outcome measures

The two outcome measures included survey opened and survey completed. An opened survey was defined as a survey linked having been used to open the survey. This was readily captured via Qualtrics distribution analytics, which records survey progress when using personalized survey links. A completed survey was defined as a survey with at least 75% of questions answered. Survey completion was only calculated among participants with an opened survey.

### Statistical analysis

Descriptive statistics (mean, median, frequencies, percentages) were calculated to characterize the study sample overall and by recruitment strategy component assignment. We used the Chi-square test to compare the proportion of individuals by consortium membership in each group. To compare the effect of each recruitment component on outcome measures, we performed multivariable logistic regression analyses controlling for consortium membership. The reference group for these models was all scenarios that did not include the component of interest. The control scenario (absence of all components) was tested as a separate model. Potential interactions between components were tested for all components and secondary regression analyses were reported stratified by the presence or absence of a component. Results are reported as adjusted odds ratios (aOR) with 95% confidence intervals (95% CI). All analyses were performed in R [43]. R package emmeans was used for assessing conditional effects when interactions were found [44, 45]. 

## Results

Surveys were collected from June to December 2023. A total of 418 (consortium A = 269; consortium B = 93; consortium C = 56) potential participants were included on distribution lists provided by consortium leadership. Eighteen entries were considered not valid participants and removed from analyses due to a bounced email (10) or personal communication indicating non-research participation in the consortium (8). Of the remaining 400 participants, 82% (341) opened the survey link and 35% (147) completed the survey (Table 1). Of those who completed the survey, participants received an average 1.5 ± 0.8 recruitment emails, had a median age of 43 [24, 70] years, 66.2% were female, 58.8% non-Hispanic White, 23.7% Asian, 6.8% Hispanic, 4.7% non-Hispanic Black, and 6% other race. The median number of years of research experience was 12 [1, 45], 60.9% had a PhD, 33.8% a MD, and 3.4% had an MD/PhD.

Overall, members of Consortium A were significantly less likely to have opened the survey link (OR 0.23, 95%CI: 0.08–0.54, *p* = 0.002) than members of the other consortia. Whereas, members of Consortium B were significantly more likely to complete the survey (OR 2.16, 95% CI: 1.10–4.30, *p* = 0.027) than members of the other consortia. There were no statistically significant differences in distribution of consortium membership or email validity between recruitment strategy assignments.

**Table 1:** Participant characteristics and outcomes by strategy assignment

Strategy	*N*	Opened % (*n*) ^a^	Completed^b^ % (*n*) ^a^
Total	400	85.0 (340)	43.2 (147)
1: Control	25	88.0 (22)	18.2 (4)
2: $1000	26	88.5 (23)	34.8 (8)
3: $50	27	85.2 (23)	30.4 (7)
4: $50, $1000	27	88.9 (24)	41.7 (10)
5: Ego	27	77.8 (21)	47.6 (10)
6: $1000, Ego	24	75.0 (18)	61.1 (11)
7: $50, Ego	26	69.2 (18)	61.1 (11)
8: $50, $1000, Ego	26	80.8 (21)	66.7 (14)
9: Altru	24	83.3 (20)	55.0 (11)
10: $1000, Altru	25	96.0 (24)	33.3 (8)
11: $50, Altru	25	92.0 (23)	30.4 (7)
12: $50, $1000, Altru	26	88.5 (23)	52.2 (12)
13: Altru, Ego	25	92.0 (23)	34.8 (8)
14: $1000, Altru, Ego	21	81.0 (17)	41.2 (7)
15: $50, Altru, Ego	23	95.7 (22)	45.5 (10)
16: $50, $1000, Altru, Ego	23	78.3 (18)	50.0 (9)

^a^ Excludes participants deemed not eligible; ^b^ Of participants who opened the survey link (*N* = 340); *Note* $50 = $50 gift, $1000 = $1000 raffle, *altru* altruistic messaging, *ego* . egoistic messaging

The effect of each recruitment strategy on opening and completing the survey and the effects when considering interactions with other recruitment strategies can be found in Table 2. Considering all strategies and their interactions, when it comes to opening the survey, an altruistic appeal increased the odds (aOR 2.02, 95% CI: 1.35–2.69, *p* = 0.033), and, with a 95%CI that did not embrace the null, egoistic messaging decreased the odds (aOR 0.56, 95%CI 0.38–0.75, *p* = 0.08) of opening the survey. Significant interactions were identified. The presence of the $50 gift decreased the effect of the altruistic messaging on the odds of opening the survey, with a 95%CI that did not embrace the null (aOR 1.90, 95%CI 1.01–2.79, *p* = 0.170 vs. aOR 2.15, 95%CI 1.15–3.15, *p* = 0.098). The $1000 raffle financial incentive decreased the effect of altruistic messaging on the odds of opening the survey (raffle present: aOR 1.48, 95%CI 0.81–2.16, *p* = 0.391 vs. raffle absent: aOR 2.77, 95%CI 1.45–4.09, *p* = 0.032). Presence of the $1000 raffle financial incentive further decreased the effect of egoistic messaging on the odds of opening the survey (raffle present: aOR 0.34, 95%CI 0.19–0.50, *p* = 0.019 vs. raffle absent: aOR 0.92, 95%CI 0.48–1.36, *p* = 0.862). On the other hand, the presence of egoistic messaging increased the effect of altruistic appeal messaging on the the odds of opening the survey (egoistic present: aOR 2.74, 95% CI: 1.52–3.96, *p* = 0.023 vs. egoistic absent: aOR 1.49, 95%CI 0.76–2.22, *p* = 0.409).

**Table 2:** Effect of recruitment strategy on opening and completing survey

			Stratified for strategy interaction: component present/component not present							
			$50 gift		$1000 raffle		Altruistic		Egoistic	
	**aOR**	**95% CI**	**aOR**	**95% CI**	**aOR**	**95% CI**	**aOR**	**95% CI**	**aOR**	**95% CI**
*Opened Survey*										
$50 Gift	1.00	0.67–1.32	-		†		1.06	0.50–1.62	†	
							0.94	0.57–1.30		
$1000 Raffle	0.90	0.61–1.20	†		-		0.66	0.31–1.01	0.55	0.31–0.79
							1.23	0.75–1.71	1.48	0.76–2.20
Altruistic	2.02**	1.35–2.69	1.90	1.01–2.79	1.48	0.81–2.16	-		2.74**	1.52–3.96
			2.15*	1.15–3.15	2.77**	1.45–4.09			1.49	0.76–2.22
Egoistic	0.56*	0.38–0.75	†		0.34**	0.19–0.50	0.76	0.36–1.16	-	
					0.92	0.48–1.36	0.41**	0.25–0.58		
*Completed Survey*										
$50 Gift	1.35	1.04–1.66	-		†		†		1.54	1.03–2.05
									1.18	0.80–1.56
$1000 Raffle	1.37	1.05–1.69	†		-		1.12	0.76–1.48	1.34	0.90–1.78
							1.68*	1.12–2.24	1.40	0.95–1.85
Altruistic	0.96	0.74–1.18	†		0.78	0.53–1.03	-		0.53*	0.35–0.70
					1.18	0.79–1.56			1.73*	1.17 –2.29
Egoistic	1.81**	1.39–2.23	2.06**	1.40–2.72	1.76*	1.19–2.33	1.00	0.68–1.32	-	
			1.58	1.05–2.11	1.85*	1.24–2.46	3.29***	2.19–4.37		

*Note* aOR adjusted for consortium affiliation; *p ≤ 0.1 **p ≤ 0.05 ***p < 0.001; † = interaction not close to significant (*p* > 0.1)

When considering completing the survey once opened, receiving only control messaging decreased the odds (aOR 0.27, 95% CI: 0.12–0.43, *p* = 0.022) and the receipt of egoistic messaging increased the odds (aOR 1.81, 95%CI: 1.39–2.23, *p* = 0.010) of completing the survey. Significant interaction effects were observed. The presence of the $50 gift increased the effect of egoistic messaging on the odds of completing the survey (gift present: aOR 2.06, 95%CI 1.40–2.72, *p* = 0.023 vs. gift absent: aOR 1.58, 95%CI 1.05–2.11, *p* = 0.167). There was a significant interaction effect between the altruistic appeal and egoistic messaging strategies for the survey completion outcome, where the presence of both strategies diminished effects of both the altruistic appeal, with a confidence interval that did not embrace the null, (egoistic present: aOR 0.53, 95% CI 0.35–0.68, *p* = 0.053 vs. egoistic absent: aOR 1.73, 95%CI 1.14 − 2.21, *p* = 0.089) and egoistic messaging (altruistic present: aOR 1.00, 95%CI 0.68–1.32, *p* = 0.995 vs. altruistic absent: aOR 3.29, 95%CI 2.19–4.437, *p* < 0.001).

There were no significant three- or four-way interactions. The effect of recruitment strategy on opening or completing the survey did not significantly vary by consortium membership.

## Discussion

Using a MOST strategy, this is one of the first studies evaluating the effectiveness of recruitment strategy components for engaging health researchers in an online survey. Overall, at approximately 37%, this study had an approximately average response rate for an online survey [8]. However, this response rate represents an above average rate as compared to multiple studies recently performed among researchers [9–19, 46]. As hypothesized, in this study we found that recruitment strategies including intrinsic motivational messaging were more likely to have an impact on health researchers’ likelihood of opening and completing an online survey as compared to extrinsic motivators. The inclusion of small-to moderate sized monetary incentives had minimal impact on participation, possibly indicating that health researchers are not primarily motivated to participate in research by extrinsic factors due to their pro-social motivations and high number of competing priorities. Egoistic and altruistic appeal messaging, however did not have consistent effects on both the opening and completion of the online survey suggesting additional complexity in health researcher participation motivations requiring further investigation. This research informs the best practice of using messaging when trying to engage researchers as research participants, adding an important yet overlooked component in meta-research evaluation.

The recruitment strategies using messaging intended to generate intrinsic motivation had a greater impact on survey participation than extrinsic motivation strategies, which is not entirely consistent with research in the general population [37]. Nevertheless, incentives have been found to undermine motivation in survey response in studies where intrinsic motivation is already high [47]. The two types of intrinsic motivation tested, altruistic and egoistic messaging, however, had disparate and inconsistent impact on both the likelihood of opening and of completing the survey. Highlighting the potentially complicated nature of motivation in survey response rates and emphasizing the need for more in-depth investigation of intrinsic motivation within the health researcher population. Future qualitative inquiry may help understand how these domains may impact engagement in survey research differently.

As observed among other college-educated professionals, [39] an altruistic appeal had a positive effect on the likelihood of health researchers opening the survey link. This effect, however did not remain significant for survey completion among those who had opened the survey link. The egoistic messaging—which has also been previously observed to have a positive impact on response rates [48]—however, had an opposite pattern of effect. In our study, the egoistic messaging significantly decreased the likelihood of opening the survey, however, among those who did open the survey, the egoistic messaging increased the likelihood of survey completion. The survey included in this study was relatively high burden; taking approximately 20 min to complete. This high burden may account for the high drop off rate between opening and completing the survey. This may have created a dynamic where an initial altruistic motivation was not strong enough to overcome the high time burden. The egoistic messaging, however, may have initially filtered out more individuals not interested in the survey topic, leaving those with an interest more likely to complete the survey. This suggests that different intrinsic motivational types may be more appropriate for low and high burden studies. More research is needed to understand the circumstances under which each motivational type is more impactful and whether alternative messaging phrasing will alter the observed associations.

When both included as components in the recruitment strategy, the altruistic and egoistic messaging created a unique set of interactions. With regard to opening the survey link, the positive effect of the altruistic messaging was strengthened by the presence of the egoistic messaging and the negative effect of the egoistic messaging was rendered no longer significant when paired with the altruistic messaging. This suggests that including both types of motivation together may increase the likelihood of an initial interest in opening the survey. The presence of both messaging types, however, did not interact similarly to impact survey completion and including both together lessened the impact of egoistic messaging on survey completion and generated a negative impact of altruistic messaging on completion. Interestingly, the control strategy, where no direct motivation (intrinsic nor extrinsic) was presented, individuals were not significantly affected for choosing to open the survey, however those individuals were significantly less likely to complete the survey. Due to the antagonistic effects of egoistic and altruistic messaging on survey completion, it may be appropriate to initiate recruitment with an altruistic appeal. Individuals’ engagement driven by the different motivation types may interact with other unmeasured factors such as tolerance for survey burden or personal interests. Thus, future research may consider using adaptive recruitment designs to provide a different recruitment strategy if a participant is a non-responder, or does not complete the survey.

The minimal response to monetary incentives is consistent with prior research among health researchers, [46] but contrary to prior research in other populations, which has shown a positive correlation between survey participation and the provision of larger monetary incentives in the general population [37]. Even small monetary incentives in, for example, physician mailed surveys were found to be effective [49]. However, our study population was slightly less than half physicians (i.e. have an MD) and thus may indicate that health researchers (whether MD or PhD) are likely to have different work-related responsibilities and differing motivations for participation than their clinician-only counterparts. At $50 and $1,000, the monetary incentives offered in this study could be considered “larger” among many populations, however, it is possible that within this potentially higher-earning population (i.e. clinicians, university faculty, etc.)—who may also be more pressed for time—a higher monetary reward may be required to incentivize participation or may not impact their engagement. While additional research is needed to understand the effective value where a motivating monetary threshold exists, it also raises the concern of budgetary feasibility of relying on even higher monetary incentives to recruit researchers. Indeed, even strategies that provide the potential for a significantly larger monetary reward, but at a feasible cost to the study (i.e. raffles), have not been successful among similar populations, with even raffles as large as $5,000 found to have minimal effect on survey participation among clinicians and college educated professionals [39, 50]. Similarly, while shown to yield superior response rates compared with conditional cash incentives paid after physicians respond to a survey, [51, 52] due to resource and logistical constraints, up-front unconditional cash incentives were not tested in this analysis. Further research comparing varying levels, and different modalities, of monetary incentives is needed to understand their full impact on engagement in this population.

Furthermore, it is possible that providing monetary incentives could actually undermine motivation to open a survey link among some health researchers when included alongside strategies of intrinsic motivation. Concordant with previously observed phenomena in behavioral research, [47, 48, 53] the inclusion of a $1,000 raffle in the recruitment strategy eliminated the significant positive impact of including altruism messaging and further increased the negative effect of egoistic messaging on the odds of opening the survey link. This effect, however, was not similarly observed for the rate of survey completion after the survey link was opened. Evidence about the impact of raffles has been mixed in the literature, however, direct incentives generally are more effective that the sweepstakes approach for boosting survey response [54]. 

### Limitations

This study had several limitations. First, the choice of email distribution led to some messages being flagged by spam filters depending on organizational settings. While corrective measures were pursued (i.e. resending messages individually as opposed to from the automated distribution list) the impact of spam filtering is unknown, likely contributing to lower rates of survey receipt that likely did not occur at random. Similarly, it was not possible to track who saw the consortium-leadership-provided prenotification and therefore the interaction of that notification with each strategy component is not known. Furthermore, excluding the greeting, the text of the recruitment e-mail was held constant throughout each follow-up. Varying messages across contacts has been shown to increase response rates, [55] therefore the lack of message variation may have diminished the observed effect of the messaging scenarios on response rates as compared to other studies that use the variation method. Second, this analysis did not assess the impact of recruitment strategy components on survey efficiency. Future research may benefit from examining the impact of recruitment strategy components on time to response. Additionally, the study did not examine multiple levels of financial incentives or alternate variations of altruistic/egoistic messages. Therefore, the effect sizes observed may not be reflective of all possible types of extrinsic and intrinsic motivational recruitment strategies. Third, beyond consortium membership, participant demographics of non-responders are unknown, limiting the ability to create generalizations about the type of health researcher to whom these findings apply and made survey nonresponse bias analyses infeasible. Fourth, as national entities, the consortia included in this study were not 100% mutually exclusive and may have had some overlap in members artificially reducing survey response or providing one individual with two different recruitment strategies. While overlap was rare (< 15 known) it may have reduced observed effects. Finally, this study only used online surveys, which tend to have lower response rates and potentially have more response bias than other survey methodologies, [8] thus caution should be used when comparing the results of our findings with the existing literature of other modalities of survey research among similar populations such as mailed surveys.

## Conclusion

When using online surveys among researchers, intrinsic motivation is likely to be a greater driver of health researcher participation in survey research than extrinsic motivation. Altruistic and egoistic messaging may differentially impact initial interest and ultimate survey completion and when combined may lead to improved rates of recruitment, but not survey completion. Further research is needed to determine how to best optimize message content and whether the effects observed are modified by survey burden.

### Electronic supplementary material

Below is the link to the electronic supplementary material.

Supplementary Material 1

## Data Availability

The datasets used and analyzed during the current study are available from the corresponding author on reasonable request.

## Abbreviations

MOST: Multiphase Optimization Strategy
aOR: Adjusted odds ratio
95%CI: 95% confidence interval
