# Supplementary material for: Evaluating strategies to recruit health researchers to participate in online survey research
Source: BMC Med Res Methodol. 2024 Jul 18;24:153. doi: 10.1186/s12874-024-02275-6 (PMC11256559; doi:10.1186/s12874-024-02275-6)
Supplement: Supplementary file 1 — Supplementary Material 1 [file 12874_2024_2275_MOESM1_ESM.docx]

| **Scenario** | **Standard Messaging** | **Altruistic Messaging** | **Egoistic Messaging** | **$50 Gift** | **$1,000 Raffle** |
| --- | --- | --- | --- | --- | --- |
| **1** | 1 | 0 | 0 | 0 | 0 |
| **2** | 1 | 0 | 0 | 0 | 1 |
| **3** | 1 | 0 | 0 | 1 | 0 |
| **4** | 1 | 0 | 0 | 1 | 1 |
| **5** | 1 | 0 | 1 | 0 | 0 |
| **6** | 1 | 0 | 1 | 0 | 1 |
| **7** | 1 | 0 | 1 | 1 | 0 |
| **8** | 1 | 0 | 1 | 1 | 1 |
| **9** | 1 | 1 | 0 | 0 | 0 |
| **10** | 1 | 1 | 0 | 0 | 1 |
| **11** | 1 | 1 | 0 | 1 | 0 |
| **12** | 1 | 1 | 0 | 1 | 1 |
| **13** | 1 | 1 | 1 | 0 | 0 |
| **14** | 1 | 1 | 1 | 0 | 1 |
| **15** | 1 | 1 | 1 | 1 | 0 |
| **16** | 1 | 1 | 1 | 1 | 1 |
| **1 = element present; 0 = element absent** | | | | | |

**Supplement Table S1. Recruitment strategy scenario element combinations**

**Supplement Table S2. Recruitment strategy elements and associated recruitment email text**

| **Recruitment element** | **Email text** |
| --- | --- |
| *Standard messaging* | To better understand what individual and environmental factors contribute to successful collaborative research, we are doing a research to collect information about health researchers and their collaboration networks. You are being contacted because you are on the member list of the [consortium name].  You are currently being invited to take part in the baseline survey component of this study. The baseline survey will last approximately 15-20 minutes. |
| *$1000 raffle* | The baseline survey will last approximately 15-20 minutes and upon completion of the baseline survey you will be entered in a drawing for a $1000 check (1 in 400 chance). |
| *$50 gift card* | The baseline survey will last approximately 15-20 minutes and upon completion of the baseline survey you will be given a $50 Amazon gift certificate. |
| *Altruistic messaging* | I recently received a K01 grant that I could use your help making successful. My study is specifically focused on the experiences of researchers themselves. I want to make sure researchers from all disciplines and career levels have a voice and are represented, but—as I’m sure you know—busy academics can be some of the hardest people to pin down! For this reason, having your participation would be invaluable. I know it can be difficult to fit a study into your already busy schedule, but I hope you will be able to squeeze my study in. I really can’t do this research without you! |
| *Egoistic messaging* | We hope you find this study interesting and useful. You as a researcher are the focus of this research. You will have an opportunity to share your perspective as well learn more about yourself as you reflect on the type connections you formed in your own research collaborations. In the long run, we hope this study will improve your experiences with research consortia and other forms of research collaboration. Your response will ensure that your perspective is included so that this research can be better benefit you. |
